# A novel agarose-free, standardized generation and versatile ECM characterization of decellularized scaffolds from normal and fibrotic human lung tissue

**DOI:** 10.3389/fbioe.2026.1772891

**Published:** 2026-03-10

**Authors:** Eike B. Preuß, Lara-Jasmin Schröder, Charlotte Hähner, Jérôme A. von Bandemer, Christopher Werlein, Edith K. J. Schwarz, Stephanie Schubert, Holger Schlüter, Matthew J. Thomas, Danny D. Jonigk, Jan C. Kamp, Mark P. Kühnel

**Affiliations:** 1 Institute of Pathology, Lung Research Group, Hannover Medical School, Hannover, Germany; 2 Biomedical Research in Endstage and Obstructive Lung Disease Hanover (BREATH), German Center for Lung Research (DZL), Hannover, Germany; 3 Boehringer Ingelheim, Biberach an der Riß, Germany; 4 Institute of Pathology, RWTH Aachen University/UKA, Aachen, Germany; 5 Department of Respiratory Medicine and Infectious Diseases, Hannover Medical School, Hannover, Germany

**Keywords:** agarose-free decellularization, decellularized humanlung scaffolds, extracellular matrix, hypersensitivity pneumonitis, idiopathic pulmonary fibrosis, recellularization, semi-quantitative image analysis

## Abstract

**Background:**

The extracellular matrix (ECM) is a key regulator of tissue homeostasis and remodeling in interstitial lung diseases (ILD) such as idiopathic pulmonary fibrosis (IPF) and fibrotic hypersensitivity pneumonitis (FHP). Decellularized lung scaffolds provide a physiologically relevant platform for studying cell-matrix interactions but generating structurally intact scaffolds from fibrotic human lungs remains challenging. Many existing protocols struggle with either insufficient removal of the cells or significant disruption of certain ECM components. In addition, all of them rely on agarose-embedding for the production of thin lung slices.

**Methods:**

Tissue samples were decellularized using apoptosis inducers (Camptothecin, Raptinal) and a mild detergent (SB-10). Standardized decellularized lung scaffolds (SDLS) were generated using a gelatin-based temporary cutting support structure, which is completely removable prior to recellularization. Decellularization and preservation of the ECM was validated by DAPI nuclear counts and semi-quantitative image analysis of collagen (picro-sirius red under polarization) and elastin (elastic Verhoeff-Van Gieson (EVG)). For functional testing, SDLS were repopulated with primary human lung fibroblasts (CD90^+^CD31^−^CD45^−^) and assessed for colonization, viability (WST-1 assay), and cytotoxicity (LDH assay).

**Results:**

The protocol removed all nuclei (>99.8%) in both fibrotic and non-fibrotic lung tissue while preserving structural integrity and the major ECM components (collagen and elastin). The novel semi-quantitative analysis proved to be accurate and versatile for the analysis of multiple ECM components within one sample. Overall, both Collagen (fresh 15.5% ± 4.3 vs. decellularized 16.7% ± 4.4) and elastin (fresh 10.0% ± 3.7 vs. decellularized 9.8% ± 3.4) remained stable post-decellularization. Fibroblasts successfully colonized SDLS, with non-fibrotic scaffolds showing pronounced contraction, whereas fibrotic scaffolds retained their dimensions. Even though fibroblasts seeded on SDLS showed significantly lower viability than those seeded on plastic plates, cytotoxicity 1 day after repopulation was not increased and remained acceptable.

**Conclusion:**

We present a reproducible, agarose-free, apoptosis-assisted method for the generation of residue-free and standardized decellularized lung scaffolds from normal and fibrotic human tissue. The novel semi-quantitative histological analysis allows the investigation of multiple ECM components from a single sample. The SDLS platform preserves key ECM components and supports controlled recellularization enabling physiologically relevant studies of matrix-cell interactions and therapeutic screening in ILD models.

## Graphical Abstract

**Figure:**
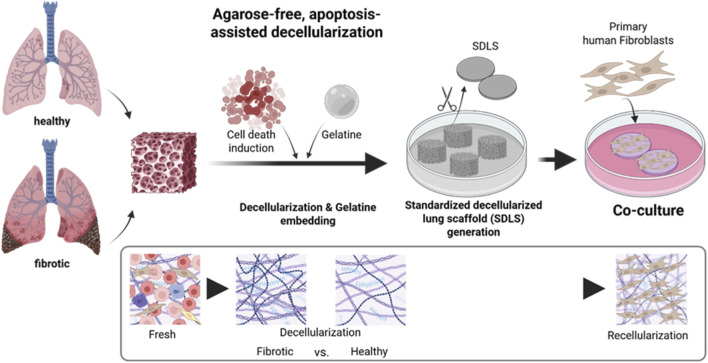

## 1 Introduction

Fibrotic remodeling of the ECM is a central driver of organ failure across multiple systems including the lung (Karsdal et al., 2017; Rockey et al., 2015). In IPF and FHP, progressive accumulation of myofibroblasts, which produce excessive amounts of ECM, results in the irreversible loss of respiratory function (Raghu et al., 2011; Lederer and Martinez, 2018; Thannickal et al., 2004; Sivakumar et al., 2012; Noble et al., 2012; Costabel et al., 2020). While IPF is typically characterized by epithelial injury and aberrant fibroblast activation with limited inflammation, hypersensitivity pneumonitis (HP) in its earlier stages is considered a primarily immune-mediated inflammatory process with secondary fibrosis (Costabel et al., 2020; Selman et al., 2001; Morell et al., 2013). At the end stage, however, both disorders show similar histopathological features such as fibroblastic foci, massive deposition of ECM, and the destruction of the original lung architecture (Morell et al., 2013; Wuyts et al., 2013; Ley et al., 2011; Tschumperlin et al., 2018; Wang et al., 2017). These biomechanical and biochemical alterations not only impair lung compliance but also provide instructive signals that further perpetuate fibroblast activation (Klingberg et al., 2013; Parker et al., 2014; Herrera et al., 2018).

The lung ECM is a complex 3D network composed of fibrillar collagens (predominantly type I and III), elastin, proteoglycans, and glycoproteins, which together maintain tissue architecture, mechanical properties, and signal transduction (Burgstal et al., 2017; Frantz et al., 2010; Faffe and Zin, 2009). In fibrotic diseases, pathological stiffening of the ECM generates a positive feedback loop of myofibroblast activation and further ECM deposition (Klingberg et al., 2013; Parker et al., 2014; Herrera et al., 2018). Understanding how disease-specific ECM alterations influence resident cell phenotypes is therefore essential for elucidating ILD pathogenesis and developing targeted therapies.

Multiple *in vitro* and *ex vivo* approaches exist to study ECM-cell interactions, each with inherent limitations. 2D monolayer culture on plastic offers reproducibility but exposes cells to supraphysiological stiffness leading to artificial activation states (Discher et al., 2005; Wells, 2008). Hydrogel-based systems permit stiffness tuning but lack the biochemical complexity and microarchitecture of diseased ECM (Burgstal et al., 2017). Furthermore, precision-cut lung slices (PCLS) preserve native ECM and cellular compartments but cannot isolate ECM-specific effects from cell-cell interactions (Ra et al., 2022; Neuhaus et al., 2017). Lastly, animal models such as bleomycin-induced fibrosis offer *in vivo* context but are not able to reflect the chronic and progressive character of fibrotic ILDs (Temann et al., 2017; Liu et al., 2017).

These constraints have led to increased use of decellularized human ECM scaffolds, which retain native biochemical composition and architecture, while allowing controlled recellularization (Moeller et al., 2006; Gilpin and Yang, 2017). Furthermore, decellularized ECM (dECM) platforms allow selective recellularization with defined cell populations enabling direct assessment of ECM-driven phenotypes (Burgstal et al., 2017; Moeller et al., 2006; Gilpin and Yang, 2017; Booth et al., 2012). Previous work has shown that fibrotic lung dECM can induce myofibroblast activation in the absence of the profibrotic master-regulator TGF-β, underscoring the ECM’s instructive capacity (Tschumperlin et al., 2018; Klingberg et al., 2013; Parker et al., 2014; Herrera et al., 2018; Burgstal et al., 2017; Frantz et al., 2010; Faffe and Zin, 2009; Discher et al., 2005; Wells, 2008; Ra et al., 2022).

However, most existing lung decellularization protocols struggle with either insufficient removal of cells or significant disruption of certain ECM components caused by harsh detergents (e.g., SDS, Triton X-100) (Wallis et al., 2012; Song et al., 2021; Gilbert et al., 2006), potentially affecting cell behavior in downstream experiments. These effects are particularly problematic in end-stage fibrotic tissue, where dense ECM impedes reagent penetration, necessitating longer and harsher decellularization protocols thereby increasing the risk of structural damage. In addition, all current protocols rely on agarose-embedding for the production of thin lung slices, introducing a foreign and non-removable substance to the system (Neuhaus et al., 2017; Temann et al., 2017; Booth et al., 2012; Koziol-White et al., 2024; Leiby et al., 2022).

To address these challenges, we aimed to utilize a previously introduced apoptosis-assisted protocol for the effective but gentle decellularization of rat lung tissue (Song et al., 2021) and adapted for normal as well as fibrotic human lung tissue to enable efficient lung tissue decellularization, preservation of ECM components, and functional recellularization capacity. This platform is intended to offer a residue-free, standardized, and pathophysiological relevant system for studying matrix-cell interactions in ILD for future perspective.

## 2 Materials and methods

### 2.1 Patient cohort and tissue collection

Human lung tissue was obtained from patients undergoing lung transplantation or lung tissue resection at Hannover Medical School. All donors provided written informed consent (DZL Informed Consent V2.0/3.0) and the study was approved by the Hannover Medical School Ethics Committee (ethic vote 8867_BO_K_2020). Clinical and demographic details are provided in Supplementary Table S1. The sample set included tissue from 29 patients in total. In detail, the fibrotic cohort consisted of 6 IPF (0% woman; mean ± SD age 61 ± 1 year), 10 FHP (10% woman; 61 ± 3 years), and 2 unclassified cases with usual interstitial pneumonia (UIP) pattern (50% woman; 66 ± 1 year). The cohort of non-fibrotic resections comprised 6 with normal histology and 5 with mild or moderate emphysema (45% women; 57 ± 16 years). Tissues were collected as soon as possible post-surgery, usually within the first hour after explantation/resection. The quality of the tissue was evaluated macroscopically by an experienced pathologist. Pleura and larger vessels or bronchi were resected. Samples were collected from each tissue, fixed with buffered formalin, and subsequently paraffin-embedded to produce histological sections for histopathological assessment. Remaining samples were stored in ice-cold medium until further procedure.

### 2.2 Isolation, culture, and cryopreservation of primary human lung fibroblasts

Fibroblasts were isolated from approximately 1 g human lung tissue using the gentleMACS™ Octo Dissociator with heaters and the Multi Tissue Dissociation Kit 1 (Miltenyi Biotec, Cat. No. 130-110-201) according to the manufacturer’s protocol. Cell suspensions were filtered sequentially through 100 μm and 70 μm strainers (Falcon, Corning). Cells were cultured in F-medium under standard conditions (37 °C, 5% CO_2_) in T75 cultivation flasks for adherent cells. F-Medium contained DMEM, low glucose, GlutaMAX™ [+] 1 g/L D-Glucose, [+] Sodium Pyruvate, [+] phenol red (Gibco, Cat. No.: 10567014) supplemented with 10% FBS and 1% Penicillin/Streptomycin. Fibroblasts overgrew other cell types after approximately 1 week. At 70%–80% confluence, cultures were split 1:2 using 0.05% Trypsin (Bio&Sell, Cat. No.: BS.L2143).

Fibroblasts were cultivated until passage 4 and then cryopreserved in cold F-medium with 10% DMSO and a cell concentration of 1–2 × 10^6^/mL. Cells were stored at −150 °C until needed. For thawing, cryotubes were warmed for approximately 2 min at 37 °C, and rapidly transferred to a T75 flask for adherent cells containing 14 mL warm F-medium. Cells were seeded at a density of 1.5–2 × 10^6^ per T75 flask. The next day, the medium was renewed to remove DMSO and cellular debris.

### 2.3 Decellularization of lung tissue

**FIGURE 1:**
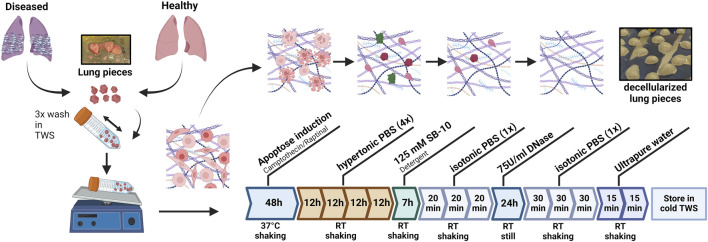
Experimental design. Decellularization workflow: Lung tissue pieces underwent apoptosis induction, followed by hypertonic washing, detergent treatment, DNase digestion, and final washes to generate standardized SDLS. Created with Biorender.

Dezellularization of human lung tissue was performed by using an adapted protocol from Song et al. (2021), with minor modifications to optimize the method for human fibrotic lung tissue. The decellularization procedure is illustrated in Figure 1. Lung tissue pieces (ca. 0.5 cm^3^) were washed twice in tissue washing solution (TWS: 1× PBS 1% Penicillin/Streptomycin/Anti-Fungi) to remove blood. Subsequently, the pieces (10–15 per 50 mL falcon tube) were incubated in 20 mL apoptosis induction medium containing DMEM/F12 [+] L-Glutamine, [+] 15 mM HEPES, [−] phenol red (Gibco, Cat. No.: 11039021), 20 μM Camptothecin (MedChemExpress, Cat. No.: HY-16560), 10 μM Raptinal (MedChemExpress, Cat. No.: HY-121320) and 1% Penicillin/Streptomycin for 48 h on a 3D shaker (37 °C, 5% CO_2_). In contrast to Song et al. (2021), who induced apoptosis in rat lung tissue using Camptothecin alone, Raptinal was added here to enhance and accelerate apoptotic signaling because Camptothecin alone did not induce sufficient apoptosis in our study. Raptinal has been shown to be a rapid and highly effective apoptosis inducer in multiple cell lines (Palchaudhuri et al., 2015). In our study, the combination of Camptothecin and Raptinal resulted in the most effective apoptosis induction (data not shown).

Following apoptosis induction, samples underwent hypertonic treatment in 25 mL 4× PBS for 48 h at room temperature with solution changes every 8–16 h to facilitate complete removal of apoptotic bodies from thicker human tissue. Tissue pieces were then exposed to 20 mL SB-10 (Sulfobetaine-10, Sigma Aldrich, Cat. No.: D4266) for 7 h at room temperature. To remove detergent residues, tissues were washed three times for 20 min each in isotonic 1× PBS. For enzymatic removal of nucleic acids, samples were subsequently incubated in 10 mL DNase solution (75 U/mL, DNAse I, Thermo Fisher scientific, Cat. No.: D4527) for 24 h at room temperature, followed by a final washing series at RT consisting of three 30 min washes in 1× PBS and two 15 min washes in ultrapure water. Fully decellularized tissue pieces were stored in PBS at 4 °C until further standardized decellularized lung scaffold generation.

In summary, the main adaptations compared to the original apoptosis-assisted protocol by Song et al. (2021) include (i) addition of the rapid apoptosis inducer Raptinal in combination with Camptothecin and (ii) use of human instead of rat lung tissue. These modifications were introduced to ensure efficient apoptotic cell clearance and preservation of ECM integrity in dense human fibrotic tissue.

### 2.4 Generation of SDLS

Decellularized lung pieces were incubated in a 50 mL falcon tube with 25 mL of 10% (w/v) liquified gelatin-preparation (Sigma Aldrich, Cat. No.: G1890) at 37 °C for 30 min on a 3D shaker, transferred to 6-well plates with 5 mL gelatin and 4 pieces per well, and shaken for another 30 min at 37 °C. Plates were then stored overnight at 4 °C to harden the gelatin. Using an 8 mm biopsy punch, cylinders were punched out and sectioned into 500 μm thick slices with an Alabama Research & Development MD6000 Tissue Slicer. Plates containing the SDLS in cold 1× PBS 1% Penicillin/Streptomycin were stored at 4 °C until further procedure.

### 2.5 Flow cytometry

Fibroblasts (500,000 cells; passage 7) were resuspended in 100 μL MACS buffer (0.5% (w/v) BSA, 2 mM EDTA) containing fluorophore-conjugated antibodies against CD90 (FITC Anti-human CD90 (Thy-1), clone REA897 Miltenyi Biotec, Cat. No.: 130-114-859), CD31 (APC Anti-human CD31, clone REA1028, Miltenyi Biotec, Cat. No.: 130-117-226), CD45 (VioBlue Anti-human CD45, clone REA747, Miltenyi Biotec, Cat. No.: 130-110-637) each 1:50 dilution or isotype controls (all from Miltenyi Biotec: Isotype FITC recombinant human IgG1, clone REA293, Cat. No.: 130-118-354; Isotype VioBlue recombinant human IgG1, REA293, Cat. No.: 130-117-362; Isotype APC recombinant human IgG1, REA293, Cat. No.: 130-120-709). After 20 min of dark incubation on ice, cells were washed with 2 mL MACS buffer, centrifuged and resuspended in 150 µL MACS buffer before analyzed using a MACSQuant® 10 flow cytometer.

### 2.6 Histological stainings

Fresh and decellularized tissue pieces were fixed in 3.5% formaldehyde (24–48 h), dehydrated, paraffin-embedded, sectioned at 2–3 μm, and mounted on glass slides. Sections were dewaxed in xylene and rehydrated through descending ethanol series. Standard histopathological stainings, including hematoxylin and eosin (H&E), elastic EVG, Gomori’s Green Trichrome (GG), and picro-sirius red (PSR) were performed according to established protocols at the Institute of Pathology, Hannover Medical School. For semi-quantitative image analysis, EVG staining was carried out without Weigert’s hematoxylin iron solution.

Nuclei staining was achieved using 4,6-Diamidin-2-phenylindol (DAPI), following antigen retrieval with TRIS-EDTA buffer (pH 9.0, 98 °C, 30 min). Slices were subsequently washed in fully deionized water, 1× wash buffer and fully deionized water, each for 5 min at RT before fully air-dried. Slides were covered up with DAPI/DuraTect-solution and stored at 4 °C in the dark until evaluated by fluorescence microscopy (APX100, Olympus Evident, Hamburg, Germany).

### 2.7 TUNEL *in situ* apoptosis assay

Apoptotic cells were detected using a one-step TUNEL kit (Elabscience, Cat. No.: E-CK-A325) according to the manufacturer’s protocol. Nuclei were counterstained with DAPI/DuraTect solution (ZytoVision, Cat.No.: MT-0008-0.8). Successful staining was visually evaluated with a fluorescence microscope (APX100, Olympus Evident) and analyzed using Fiji.

### 2.8 Image acquisition and analysis

Images were acquired using an optical and a fluorescence microscope (BX53F2 and APX100, Olympus Evident). Fluorescent signal was recorded in the DAPI and the GFP channel to differentiate between the true DAPI signal and the autofluorescence of the connective tissue fibers. Images were taken from randomly selected locations of the tissue section. Pictures were taken at ×100 magnification at two randomly selected locations on each tissue section. Analysis of the images was performed in Fiji/ImageJ using specific color thresholds for each analysis and automated macros for batch processing. A more detailed description of the image acquisition and analysis is provided in the Supplementary Methods. Data from technical replicates were averaged and subjected to statistical analysis with GraphPad Prism v10.

### 2.9 Repopulation of SDLS

Before seeding, gelatin in the SDLS was liquified by shaking incubation at 37 °C for 1 h. Subsequently, liquid gelatin was removed and the SDLS were washed 2 times with 1 mL TWS (1xPBS 1% PSA) per SDLS at 37 °C for 30 min 24-well suspension plates were coated with 500 µL anti-adherence solution per well (StemCell Technologies, Cat. No.: 07010) as stated in the manual to prevent fibroblast adhesion to the plastic surface. After three washing steps with 1 mL PBS/well, each well was filled with 200 μL of co-culture (CC) medium, containing DMEM (Gibco, Cat. No.: 11995065) with 4 mM GlutaMAX Supplement, 1 mM Sodium pyruvate, 1 g/L D-Glucose, 0.2 mM L-Ascorbic acid 2-phosphate, 0.4% FBS and 1% Penicillin/Streptomycin. A de-gelatinized SDLS was carefully transferred to each well of the anti-adherence solution coated 24-well plate. At the same time 500,000 fibroblasts of each batch were characterized by CD90, CD31, CD45 flow cytometry analysis as previously described. A cell suspension of 200,000 fibroblasts in 100 µL CC-medium was seeded onto each SDLS that was pre-incubated in 200 µL of CC-medium at 37 °C for 1 h. To evenly distribute the fibroblasts inside the SDLS, the plates were shaken under standard culture conditions for 15–16 h. The next day, the plates were removed from the shaker and incubated at rest to allow the fibroblasts to settle before any further procedure. Successful colonization of the SDLS without cells growing on the plates was checked under the microscope.

### 2.10 Viability and cytotoxicity assays

Co-culture viability was assessed in 24-well plates using two assays: water-soluble tetrazolium (WST)-1 reagent (Sigma Aldrich, Cat. No.: 5015944001) for metabolic activity and lactate dehydrogenase (LDH)-assay (Roche, Cat. No.: 11644793001) for cytotoxicity. For WST-1, the reagent was freshly diluted 1:10 in CC-medium. Each well contained one repopulated SDLS. After removing the CC-medium, 250 µL working solution was added and incubated for 1 h under standard conditions (37 °C, 5% CO_2_). Then, 100 µL supernatant was transferred in duplicates to a flat-bottom 96-well plate with blanks (working solution without sample) and measured at a wavelength of 450 nm using a plate reader (reference 630 nm; Synergy 2, BioTek). To standardize LDH measurement, culture medium was refreshed 24 h prior to the assay. For LDH, 50 µL supernatant per well (duplicates) was compared to a positive control (1% Triton X-100 for 45 min) and its 1:5 and 1:10 dilutions in CC-medium to keep absorbance in range. The detergent 1% Triton X-100 lysed all cells thereby releasing the total existing LDH of the fibroblasts. The working solution was prepared according to the manufacturer’s instructions, incubated for 20 min at RT in the dark, and measured at 492 nm (reference 630 nm) using a plate reader. Unconditioned medium served as blank. Cytotoxicity was calculated as % LDH release relative to the positive control (dilution values multiplied by respective factors).

### 2.11 Statistical analysis

Data were analyzed with GraphPad Prism v10. Unpaired t-tests: fresh vs. decellularized samples. One-way or two-way ANOVA with Tukey’s *post hoc* test: multiple comparisons in viability assays. Statistical significance was set to p < 0.05. Data are expressed as mean ± SD.

## 3 Results

### 3.1 Efficient cell removal and structural preservation following decellularization

**FIGURE 2:**
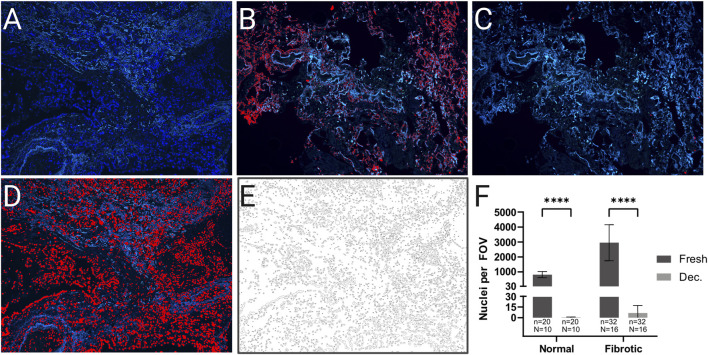
Decellularization removes cell nuclei. Analysis of cell nuclei removal from decellularized lung tissue **(A–F)**: Merged channel (DAPI and GFP) images of fresh and decellularized (decellularized) tissue sample sections with different color-threshold settings. **(A)** Image of a fresh tissue section with a color-threshold set too low for adequate DAPI signal detection (Hue: 150–255). **(B)** Image of a decellularized tissue section with a color-threshold set too high, resulting in non-specific DAPI signal detection (Hue: 170–255) (red overlay). **(C)** Image of a decellularized tissue section with a color-threshold set for specific and adequate DAPI signal detection (Hue: 160–255). **(D)** Image of a fresh tissue section with a color-threshold set for specific and adequate DAPI signal detection (Hue: 160–255) (red overlay). **(E)** Outlines of the counted DAPI signals from **(D)**. **(F)** Quantification of the nuclei counted per field of view (FOV) in fresh and decellularized normal and fibrotic tissue sample sections. The means of the technical replicates (n) were calculated and used for data plotting and statistical analysis with GraphPad Prism v10. Data are expressed as means ± SD. ∗∗∗∗ *p* < 0.0001. n, number of FOVs (field of view); N, number of lungs. Original magnification: ×100 **(A–E)**.

**FIGURE 3:**
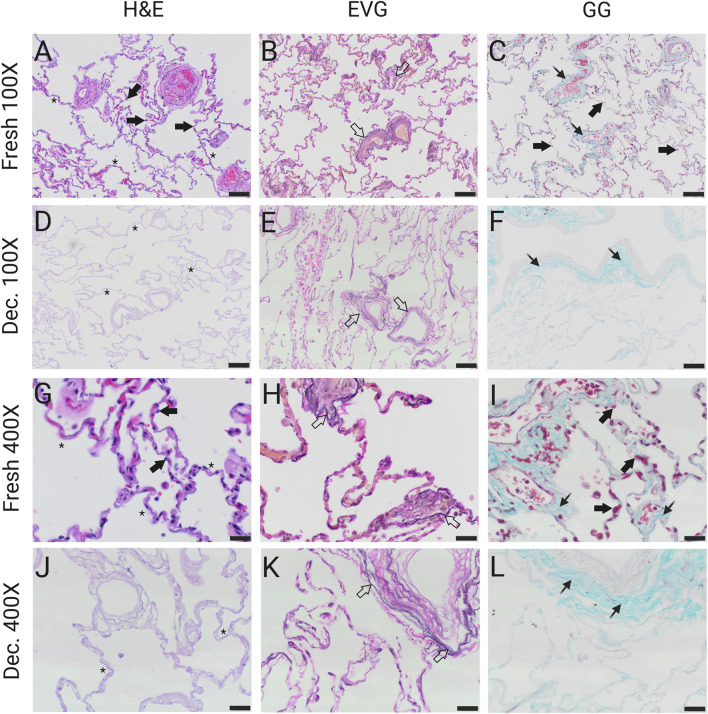
Decellularization maintains lung tissue ECM integrity. Qualitative assessment of structural integrity before and after decellularization of human lung tissue. The structural integrity was assessed using different histopathological staining methods. H&E stains cell nuclei blue and the cytoplasm, collagen fibers, and erythrocytes are stained red with varying intensity. EVG stains nuclei and elastic fibers dark purple to black and collagen red. Gomori’s Green Trichrome (GG) stains collagen teal and cells, erythrocytes, and fibrin pink to red. Arrows indicate cell nuclei, hollow arrows indicate elastic fibers, spiky arrows indicate collagen, and asterisks indicate alveolar septa. **(A–C)** Low-power magnification of unprocessed (fresh) lung tissue in H&E **(A)**, EVG **(B)** and GG **(C)** stain. **(D–F)** Low-power magnification of decellularized (decellularized) lung tissue in H&E **(D)**, EVG **(E)** and GG **(F)** stain. **(G–I)** High-power magnification of fresh lung tissue in H&E **(G)**, EVG **(H)** and GG **(I)** stain. **(J–L)** High-power magnification of decellularized lung tissue in H&E **(J)**, EVG **(K)** and GG **(L)** stain. Scale bars: 100 μm **(A–F)**; 20 μm **(G–L)**. Original magnifications: ×100 **(A–F)**; ×400 **(G–L)**.

The success of a decellularization protocol is defined by the efficient removal of cellular components while maintaining the structure and components of the ECM (Crapo et al., 2011). The induction of apoptosis was verified using the TUNEL *in situ* apoptosis assay. Interestingly, while normal (non-fibrotic) lung tissue samples exhibited a significant increase in apoptotic cells upon treatment compared to untreated controls, fibrotic lung tissue samples displayed comparable levels of apoptosis irrespective of incubation with the apoptosis inducers (Supplementary Figure S1). A common and widely accepted indicator of successful removal of cellular material is the evaluation of the nuclear material remaining in the tissue (Crapo et al., 2011). Here, cell removal was evaluated by DAPI staining and fluorescence microscopy. Images were acquired at ×100 magnification at two randomly selected locations on each tissue section, with the DAPI channel for nuclei and the GFP channel to capture connective tissue autofluorescence. Image analysis was performed on merged channels to subtract false-positive nuclear signals caused by connective tissue autofluorescence. Specific thresholds for hue (150–255), saturation (0–255), and brightness (80–255) were applied (Figures 2A–E). In total, 10 normal and 16 fibrotic (5 IPF, 9 FHP and two unspecified fibrotic lungs with UIP pattern) lung tissue samples (before and after decellularization) were analyzed. Fresh fibrotic lung tissue had approximately threefold more nuclei per field of view (FOV) than fresh normal lung tissue (fibrotic: mean ± SD, 2,950.3 ± 1,204.6 nuclei/FOV; normal: 811.8 ± 204.6 nuclei/FOV; p < 0.0001). Following decellularization, nuclei counts were reduced to 6.6 ± 10.5 in fibrotic tissue and 0.5 ± 0.5 in normal lung tissue, corresponding to >99.8% nuclear removal (p < 1 × 10^−6^ for both vs. fresh; unpaired t-test; Figure 2F). Histopathological examination revealed intact alveolar septa post-decellularization, with the absence of visible nuclei in H&E staining (Figures 3A,D,G,J). EVG staining showed preserved elastin fibers around the vasculature (Figures 3B,E,H,K), and Gomori’s Green Trichrome staining confirmed an intact collagen network without evidence of degradation (Figures 3C,F,I,L), as assessed by histological observation. These findings indicate that the protocol removes cellular material effectively while maintaining ECM microarchitecture.

### 3.2 Collagen and elastin preservation in decellularized lung tissue

**FIGURE 4:**
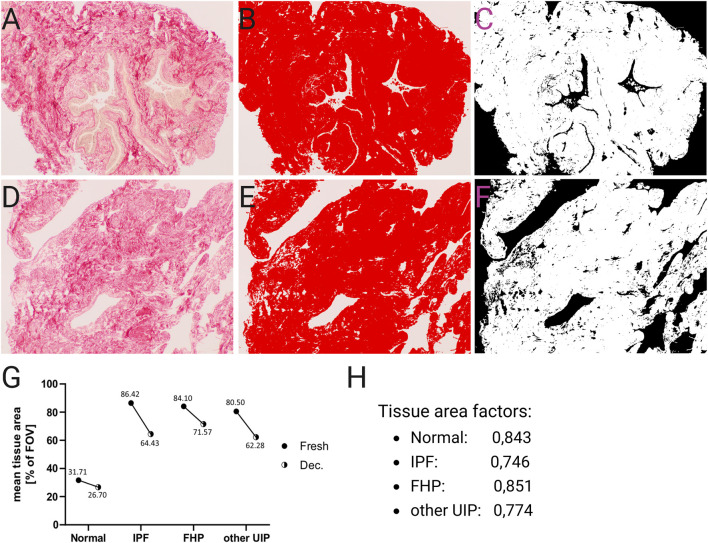
Reduction of tissue area following decellularization. Analysis and calculation of the relative tissue area in unprocessed and decellularized lung tissue samples **(A–H)**. The relative tissue area in each image was quantified and used to compensate for different densities of the tissue samples and to exclude non-tissue areas like airspaces of healthy alveoli. Additionally, a tissue area factor was calculated to compensate for the reduced tissue area resulting from the removal of cells. **(A)** Exemplary image of unprocessed (fresh) lung tissue in picro-sirius red stain. **(B)** The same field of view (FOV) as in **(A)**, where the tissue area is selected (red overlay) using specific color threshold settings. **(C)** Overlay of the signal detected in the image from **(B)**. **(D)** Exemplary image of decellularized lung tissue in pricro-sirius red stain. **(E)** The same FOV as in **(D)**, where the tissue area is selected (red overlay) using specific color threshold settings. **(F)** Overlay of the signal detected in the image from **(E)**. **(G),** Mean relative tissue area per FOV of fresh and decellularized tissue sections with different pathological backgrounds. **(H)** Tissue area factor to compensate for area reduction by the removal of cells. It is the quotient of the relative decellularized tissue area and the fresh tissue area and calculated separately for each entity. Original magnifications: ×100.

**FIGURE 5:**
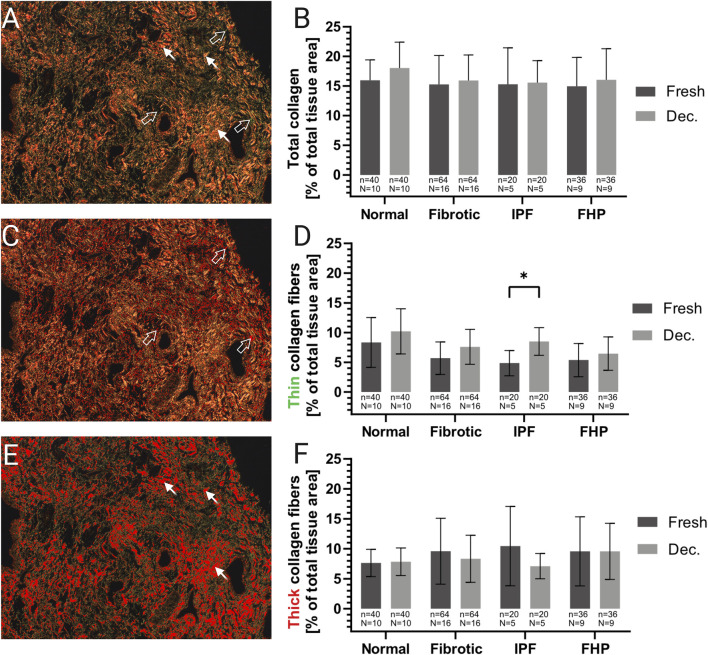
Preservation of collagen following decellularization. Quantitative analysis of collagen preservation in decellularized lung tissue samples. Relative tissue area of collagen fibers was analyzed. **(A)** Polarized-light image of a PSR stained decellularized (decellularized) tissue section. While thick collagen fibers appear in the spectrum of yellow and orange (spiky arrows), thin collagen fibers appear greenish (hollow arrows). **(B)** Mean relative (% of total tissue area) total collagen fiber area in fresh and decellularized tissue sections with different pathological backgrounds. **(C)** Polarized-light image of a PSR stained decellularized tissue section with a color-threshold set for the specific detection of thin collagen fibers (hollow arrows). **(D)** Mean relative (% of total tissue area) thin collagen fiber area of fresh and decellularized tissue sections, with different pathological backgrounds. **(E)** Polarized-light image of a PSR stained decellularized tissue section with a color-threshold set for the specific detection of thick collagen fibers (spiky arrows). **(F)** Mean relative (% of total tissue area) thick collagen fiber area of fresh and decellularized tissue sections with different pathological backgrounds. The means of the technical replicates (n) were calculated and used for data plotting and statistical analysis with GraphPad Prism v10. Data are expressed as means ± SD. ∗ p < 0.05. n, number of FOVs (field of view); N, number of lungs. Original magnifications: ×100.

**FIGURE 6:**
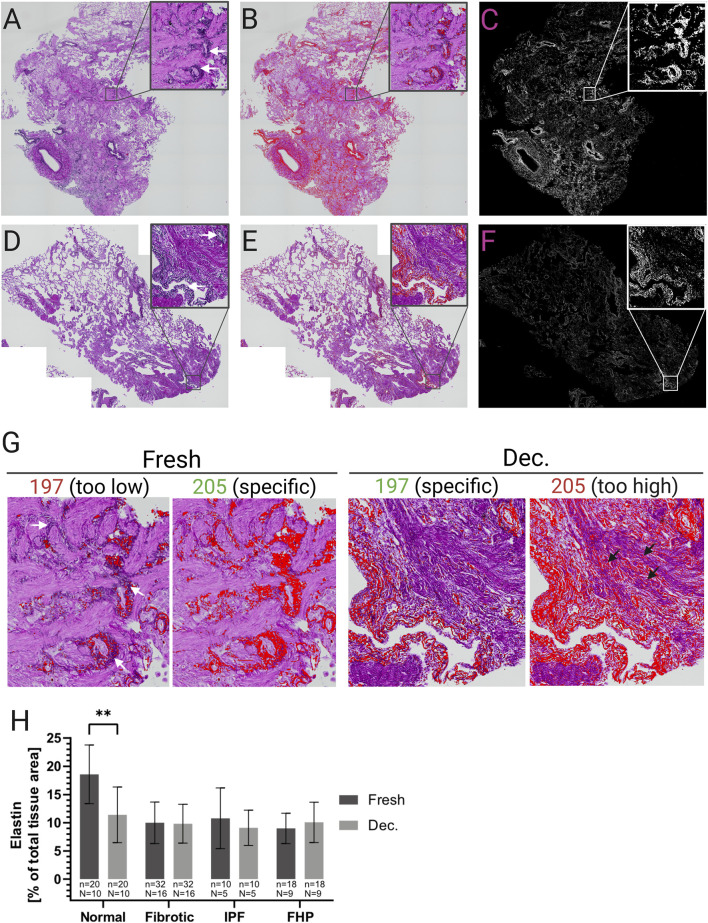
Analysis of elastin content in unprocessed (fresh) and decellularized lung tissue. The relative area of elastin was analyzed using the stitched images of the whole tissue sections, which were stained with EVG without the Weigert’s hematoxylin iron solution (nuclei staining). Elastin is colored dark purple to black (arrows). **(A)** Image of an EVG stained fresh tissue section. Insert shows a magnified area with visible elastin. **(B)** The same field of view as seen in **(A)** with a color-threshold set for the specific detection of elastin (red overlay). **(C)** Overlay of the signal detected in the image from **(B)**. **(D)** Image of a EVG stained decellularized (decellularized) tissue section. Insert shows a magnified area with visible elastin. **(E)** The same field of view as seen in **(D)** with a color-threshold set for the specific detection of elastin (red overlay). **(F)** Overlay of the signal detected in the image from **(E)**. **(G)** Exemplary images of EVG stained fresh and decellularized tissue sections with different threshold (“Hue”) settings for elastin detection. While a threshold value of 205 achieved a specific and adequate elastin detection in fresh tissue sections (red overlay), it led to a lot of non-specific signal detection of collagen in decellularized tissue sections (spiky arrows). A threshold value of 197 resulted in specific elastin detection for the decellularized tissue sections (red overlay) but was inadequate for elastin detection in fresh tissue sections (arrows). Therefore, a threshold value of 205 was used for fresh tissue sections and a value of 197 for decellularized tissue sections. **(H)** Mean relative (% of total tissue area) elastin area in fresh and decellularized tissue sections with different pathological backgrounds. The means of the technical replicates (n) were calculated and used for data plotting and statistical analysis with GraphPad Prism v10. Data are expressed as means ± SD. ∗∗ p < 0.01. n, number of whole tissue sections; N, number of lungs.

To evaluate ECM integrity after decellularization, the abundance of collagen and elastin per tissue section was examined semi-quantitatively using established standard histopathological staining methods (Figures 4–6; Supplementary Figure S2A,B), the image analysis software Fiji, and specific threshold settings (Supplementary Figure S2C–F). In total, 10 normal and 16 fibrotic (5 IPF, 9 FHP, and two unspecified fibrotic lungs with UIP pattern) lung tissue samples (before and after decellularization) were analyzed. The PSR stain was used to determine the relative area of the tissue in the bright-field image (Figures 4A–F). Bright-field images of PSR-stained tissue under polarized light allowed separate detection of thick collagen fibers, which appeared yellow-orange, and thin fibers, which displayed a green birefringence (Supplementary Figure S2B). This phenomenon was used to apply a threshold filter to measure the area of the thick (Supplementary Figures S2C,D) and thin (Supplementary Figures S2E,F) collagen fibers, which were then put in relation to the tissue area in each image. The means of the relative collagen area within the tissue samples were calculated from four images. Since decellularization reduces overall tissue density by removing of cells (Figure 4G), a tissue-area correction factor was applied to all decellularized samples to ensure accurate comparison (Figure 4H). For each tissue section, n = 4 images were analyzed with and without a polarization filter at randomly selected locations.

To assess the preservation of collagen within the decellularized tissue, semi-quantitative analysis of the lung tissue samples (10 histologically inconspicuous and 16 fibrotic lung tissues) before (fresh) and after decellularization was performed. (Figures 5A,B): Across all pathological backgrounds (normal, FHP, IPF), the mean relative total collagen area remained stable (fresh 15.5% ± 4.3 and decellularized 16.7% ± 4.4) (Figures 5A,B). Interestingly, the relative content of thin collagen fibers was slightly higher in decellularized lung tissue (mean ± SD, 8.3% ± 3.2% p < 0.03) than in fresh lung tissue (mean ± SD, 6.5% ± 2.9%) in all entities (Figures 5C,D). Notably, in IPF samples, thin collagen fiber content was significantly increased in decellularized compared to fresh tissue post-decellularization (8.5% ± 2.3% vs. 4.9% ± 2.1; p = 0.03). However, the analysis revealed no apparent reduction in thick fiber content due to the decellularization procedure in normal as well as in fibrotic lung tissue (fresh: 9.2% ± 5.0; decellularized: 8.3% ± 3.6) (Figures 5E,F). One theory is hat this modest shift might reflect detergent-induced fragmentation of thick collagen fibers into thinner fibrillar structures during decellularization as collagen denaturation upon detergent exposure has previously been reported. Such partial disassembly would reduce the proportion of thick fibers while proportionally increasing the detectable thin-fiber fraction, without representing true collagen loss.

Elastin was assessed on EVG-stained sections using analogous image analysis (Figures 6A–F; Supplementary Figure S3 featuring Fiji analysis with corresponding threshold settings. The mean relative elastin area, normalized to the total tissue area, was quantified on two whole lung sections per condition (fresh vs. decellularized) and disease entity (normal n = 10, IPF n = 5, FHP n = 9, and fibrotic lungs with UIP pattern n = 2) (Figure 6H). During image analysis, it was observed that color intensities in the stained tissue sections differed slightly between unprocessed (fresh) and decellularized samples. This variation led to either inadequate or non-specific elastin detection when identical threshold settings were applied to both conditions. Following a comprehensive assessment by a histologically highly experienced biologist and pathologist, group-specific thresholds were carefully selected. This expert-led approach ensured that the detection overlay accurately matched the actual elastin fibers across samples from different donors and entities (Figure 6G). Hence, specific thresholds were set separately for fresh (hue ≤ 205) and decellularized (hue ≤ 197) sections to accurately detect the elastin in those samples (Figure 6G). After correction for tissue area, elastin levels were preserved in fibrotic samples (fresh 10.0% ± 3.7 vs. decellularized 9.8% ± 3.4; n.s.), whereas non-fibrotic tissue exhibited a significant reduction in decellularized scaffolds (fresh 18.6% ± 5.2 vs. decellularized 11.4% ± 4.9; P < 0.01) (Figure 6H).

### 3.3 Isolation and phenotyping of primary lung fibroblasts

**FIGURE 7:**
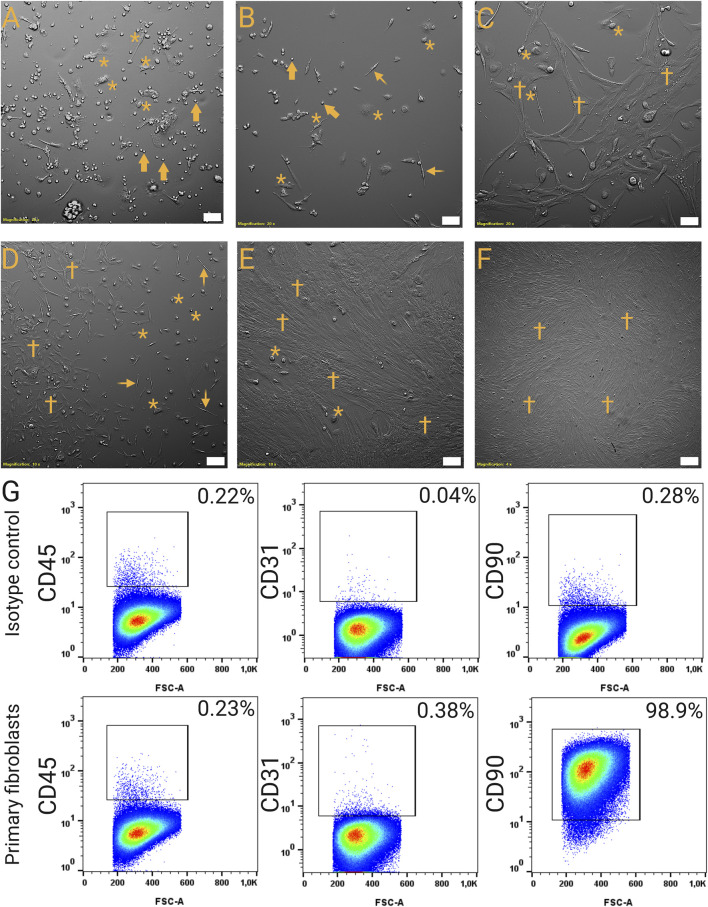
Cultivation of highly pure human primary fibroblasts from fibrotic end-stage lungs. **(A–F)** Fibroblast culture in early and late stages. **(A)** High-power magnification of a lung tissue cell isolate 24 h after tissue dissociation and seeding. Various adherent (asterisks) and non-adherent (arrows) cell types are visible. **(B)** High-power magnification of a lung tissue cell isolate 24 h after tissue dissociation and after one wash/medium change. Non-adherent cells (arrows) have been reduced significantly. Fibroblast-like (elongated) phenotypes (spiky arrows) are visible. **(C, D)** High- **(C)** and low-power **(D)** magnification of the cell culture after 7 days. Activated fibroblasts (dagger) that are increased in size start to form cluster. **(E)** Low-power magnification of the cell culture after 14 days. Fibroblasts (dagger) start to overgrow other cell types (asterisks). **(F)** Overview of a 21-day-old culture that has been split once. Fibroblasts (dagger) cover approximately 99% of the surface of the culture plate, other cell types are not visible. Scale bars: 100 μm Original magnifications: ×200 **(A–C)**; ×100 **(D, E)**; ×40 **(F)**. **(G)** Gating strategy to validate the purity of fibroblasts in passage 7 before co-culture. Top section: Isotype control: Exemplary sample of primary human fibroblasts incubated with IgG-FITC, IgG-APC and IgG-VioBlue at passage 7. Bottom section: Exemplary sample of primary human fibroblasts stained with anti-CD90 (conjugate: FITC), anti-CD31 (conjugate: APC) and anti-CD45 (conjugate: VioBlue) at passage 7. APC, Allophycocyanin; FITC, Fluorescein isothiocyanate; FSC-A, Forward scatter area; FSC-H, Forward scatter height; IgG, Immunoglobulin G; SSC-A, Side scatter area.

To confirm the suitability of the established decellularization protocol for cell culture applications, we reseeded SDLS with primary human fibroblasts, demonstrating that the scaffolds supported fibroblast attachment, colonization, and viability. Primary human fibroblasts were isolated from nine donor lungs (normal, IPF, FHP, each n = 3) and cultured for at least three passages until they reached purity. Morphologically, fibroblasts progressively overgrew other cell types between day 7 and day 21 of cultivation (Figures 7A–F). Flow cytometry directly before setting up co-cultures, confirmed a high-purity population expressing CD90 (98.1% ± 1.7%) and negative for endothelial marker CD31 and leukocyte marker CD45 (<1% each) (Figure 7G; Supplementary Table S1).

### 3.4 Recellularization of SDLS with primary human fibroblasts

**FIGURE 8:**
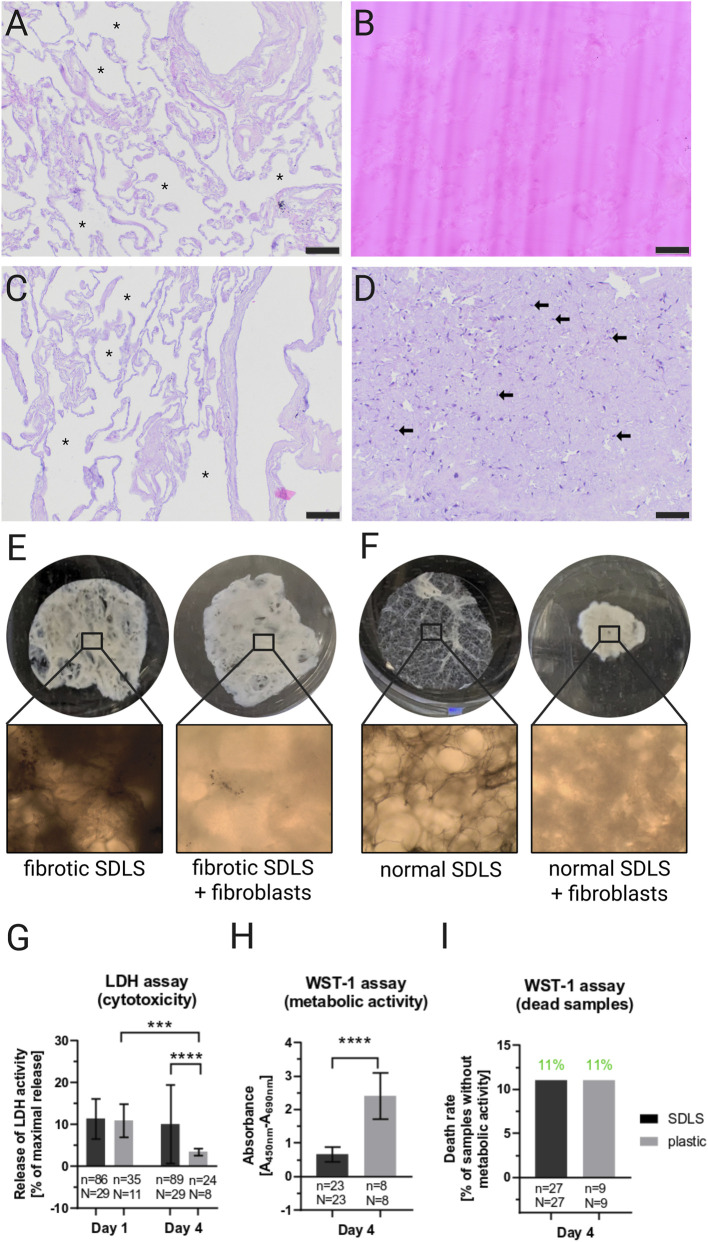
Recellularization of SDLS with human primary fibroblasts. **(A–C)** Decellularized lung tissue before and after gelatin embedding and after repopulation. Microscopic images stained with H&E **(A)** Decellularized lung tissue. Structural integrity is preserved and no cell nuclei (which would appear in dark purple) are visible. Airspaces within the alveolar network are indicated with asterisks. **(B)** Decellularized lung tissue, which is embedded in gelatin. **(C)** Standardized decellularized lung scaffold after the cutting procedure and after the gelatin was washed out of the tissue. No residues of gelatin are visible. The structural integrity is preserved. Airspaces within the alveolar network are indicated with asterisks. **(D)** Standardized decellularized lung scaffold that was repopulated with human primary fibroblasts (arrows). Cell nuclei (appear in dark purple) are scattered heterogeneously across the tissue. **(E, F)** SDLS which were generated by gelatin-based cutting support before and after colonization with human primary lung fibroblasts. **(E)** Macroscopic (top section) and microscopic (bottom section) images of a fibrotic SDLS with and without fibroblast colonization. The shape and size of the fibrotic SDLS does not change after colonization with fibroblast. **(F)** Macroscopic (top section) and microscopic (bottom section) images of a non-fibrotic/normal SDLS with and without fibroblast colonization. The diameter of the normal SDLS is significantly reduced after colonization with fibroblast. Original magnification, ×40 (bottom section). **(G–I)** Viability and cytotoxicity of SDLS-fibroblasts co-cultures. Viability was measured by the water-soluble tetrazolium (WST)-1 (for quantification of cell proliferation and viability) and LDH (for quantification of cell death and cell lysis) assays. **(G)** Results of the LDH assay measuring the cytotoxicity induced by SDLS 24 h and 4 days after colonization with human primary lung fibroblasts. **(H)** Results of the WST-1 assay measuring the metabolic activity of SDLS-fibroblast co-cultures at day 4. **(I)** The relative amount of dead samples that showed no metabolic activity as measured by the WST-1 assay at day 4. WST-1 values are shown as the corrected absorbance, and LDH values are represented as the relative amount of released LDH activity compared with the totally lysed control. For LDH assay, data is displayed following ANOVA [F(1,233) = 24.36, p < 0.0001 for difference between day 1 and day 4; F(1,233) = 11.29, p < 0.0001 for difference between SDLS and plastic] and Tukey’s multiple comparison test (shown in Figure) while data for WST-1 assays is displayed by unpaired t-tests. The means of the technical replicates (n) were calculated and used for data plotting and statistical analysis with GraphPad Prism v10. Data are displayed as means ± SD. ∗∗∗∗ p < 0.0001. n, number of repopulated SDLS; N, number of lungs.

Next, we sought to confirm that a gelatin-based cutting support is superior for subsequent repopulation due to residue-free scaffolds. In contrast to agarose, which remains as a permanent polymeric filler within the tissue and cannot be removed before seeding, the gelatin used in our protocol is thermoreversible and can be completely liquified and washed out at 37 °C prior to recellularization. This ensures that the scaffolds consist exclusively of native ECM and that no residual support material interferes with cell adhesion, migration, or nutrient diffusion. Indeed, agarose-embedded scaffolds impaired fibroblast attachment and promoted cell clustering (Supplementary Figure S4), whereas the agarose-free approach enabled the generation of structurally intact SDLS composed exclusively of ECM, which supported efficient fibroblast adhesion, and homogeneous distribution (Figures 8A–D).

Following clearance of SDLS, fibroblasts were seeded onto these previously gelatin-embedded scaffolds from the aforementioned pathological backgrounds, enabling nine different fibroblast–scaffold combinations with two biological replicates each. Upon fibroblast seeding, SDLS underwent macroscopic alteration (Figures 8E,F). Non-fibrotic SDLS contracted after fibroblast colonization, while fibrotic SDLS maintained their diameter, implying a dense and stiff nature of fibrotic lung tissue after decellularization.

### 3.5 Viability and cytotoxicity assays

Fibroblast viability and cytotoxicity were measured at day 1 and day 4 after seeding on SDLS or standard plastic culture. Initial cytotoxicity of SDLS measured after the first 24 h after colonization (11.3% ± 4.5%; n = 86) was comparable and not significantly different to the values measured of fibroblasts seeded on standard culture plate (10.9% ± 4.0%; n = 35; n.s.). However, at day 4, LDH assay values of SDLS-fibroblast co-cultures (9.38% ± 9.1%; n = 89) were significantly higher than those of fibroblasts cultured on plates (3.4% ± 0.8%; n = 27; p < 0.0001) but not significantly different from the values measured after 24 h (Figure 8G). Overall, cytotoxicity values of SDLS fibroblast co-cultures were below 15% of the detergent (1% Triton X-100) lysed positive control, not indicating any major cytotoxicity of SDLS. At day 4, viability, as measured by metabolic activity in the WST-1 assay, was significantly higher in fibroblasts on plastic (2.40 ± 0.69; n = 8 than in SDLS fibroblast co-cultures (0.66 ± 0.22; n = 23; p < 0.0001; Figure 8H). The proportion of non-viable samples (no WST-1 signal) was similar (11%) for both conditions (Figure 8I).

## 4 Discussion

The present study introduces a novel standardized, agarose-free method for the generation and versatile ECM characterization of decellularized scaffolds from physiologically healthy and fibrotic human lung tissue. The protocol is able to remove >99.8% of cell nuclei while preserving the gross architecture and structural components of the ECM. In contrast to many existing decellularization strategies, our approach relies on apoptosis induction combined with a mild zwitterionic detergent (SB-10), enabling efficient yet less disruptive cell removal, thereby preserving key ECM components such as collagen and elastin (Booth et al., 2012; Wallis et al., 2012; Gilbert et al., 2006). Importantly, the production of SDLS does not require agarose embedding. Instead, a temporary gelatin-based support is used, which can be completely removed prior to recellularization. This ensures that the resulting scaffolds are composed solely of native ECM, free of non-removable foreign material that might otherwise interfere with cell–matrix interactions (Neuhaus et al., 2017; Booth et al., 2012). Together, these refinements overcome major limitations of previous protocols and provide a residue-free, physiologically relevant platform for ILD research. The introduction of a novel semi-quantitative image analysis of stained histological sections enables the evaluation of multiple components from a single tissue sample after decellularization. This approach is resource-efficient, compatible with standard histopathological stainings, and allows both quantitative and qualitative analyses. Moreover, it can also be applied retrospectively. The platform introduced here constitutes a robust, physiologically relevant tool for studying ECM-cell interactions and for screening anti-fibrotic therapies in a disease-specific context.

### 4.1 Methodological advances over existing protocols

Perfusion-based decellularization methods have been successfully applied to whole porcine and human lungs, preserving architecture and mechanical properties, but requiring complex infrastructure and being less amenable to high-throughput applications (Ott et al., 2008; Petersen et al., 2010; Price et al., 2010; Gilpin et al., 2014). Precision-cut lung slices (PCLS) preserve native ECM and cell populations (Neuhaus et al., 2017; Temann et al., 2017), but do not permit isolation of ECM-specific effects from cell-cell interactions. Hydrogel-based systems allow tunable stiffness (Caracena et al., 2022), but lack the full biochemical and microarchitectural complexity of human fibrotic ECM. Decellularized ECM scaffolds overcome some of these limitations by retaining disease-specific biochemical cues and stiffness signatures, which have been shown to induce myofibroblast activation even in the absence of soluble profibrotic mediators (Tschumperlin et al., 2018; Parker et al., 2014; Marinković et al., 2012). Our approach expands on earlier apoptosis-based decellularization studies in other tissues (Gilpin and Yang, 2017; Song et al., 2021; Cornelison et al., 2018) by applying it to human lung tissue from both healthy and fibrotic sources including IPF and FHP. Notably, the method proved effective even in dense, collagen-rich fibrotic matrices, where reagent penetration is often limited, and risk of structural damage is high (Gilpin and Yang, 2017).

In direct comparison, previous lung slice generation protocols have often embedded tissue in agarose to provide cutting support (Neuhaus et al., 2017; Temann et al., 2017; Booth et al., 2012). Agarose-based tissue embedding leaves polymeric residues that can interfere with cell adhesion, cell-matrix interaction, signaling, and nutrient diffusion (Ulrich et al., 2011; Johnson et al., 1996). Our method replaces agarose with gelatin embedding, which can be fully removed prior to recellularization, ensuring direct cell-ECM contact in functional assays (Figure 8). A major challenge in fibrotic lung tissue is incomplete penetration of detergents due to dense collagen networks and increased crosslinking (Karsdal et al., 2017; Wijsenbeek et al., 2022). Similarly, conventional decellularization protocols often rely on strong detergents such as sodium dodecyl sulfate (SDS) or Triton X-100, which can cause loss of essential ECM proteins (collagen, elastin, glycosaminoglycans) and disrupt ultrastructural integrity, potentially impairing downstream cell-matrix interactions (Wallis et al., 2012; Song et al., 2021; Gilbert et al., 2006). In contrast, our method combines apoptosis induction using Camptothecin and Raptinal with mild detergent treatment (SB-10), enabling >99.8% nuclear clearance while preserving key proteins of the ECM (collagen and elastin). By incorporating an apoptosis induction step using Camptothecin and Raptinal prior to detergent exposure, endogenous cellular dismantling facilitates subsequent detergent and nuclease penetration, improving decellularization efficiency (Song et al., 2021). This two-stage approach avoids prolonged exposure to chemical agents that may damage ECM proteins (Gilbert et al., 2006) as supported by our semi-quantitative collagen and elastin analyses.

### 4.2 ECM preservation and implications for functional studies

Fibrotic ECM is increasingly recognized as an active participant in disease progression, influencing fibroblast phenotype through altered stiffness, crosslinking, and biochemical composition (Klingberg et al., 2013; Parker et al., 2014; Herrera et al., 2018; Burgstal et al., 2017; Frantz et al., 2010; Faffe and Zin, 2009; Discher et al., 2005; Wells, 2008; Neuhaus et al., 2017; Temann et al., 2017; Liu et al., 2017; Moeller et al., 2006). The interstitial matrix of the lung is largely composed of type I and III collagens and elastin as the major contributors to the 3-dimensional structure (Burgstal et al., 2017; Frantz et al., 2010). The aforementioned proteins are also found to be elevated in fibrotic lung tissue (Jessen et al., 2021; Hansen et al., 2016). The preservation of these ECM components is essential to enable physiologically relevant cell-ECM interaction studies in the context of fibrosis. As assessed by semi-quantitative image analysis of histological sections, our protocol greatly preservedcollagen fibersacross all disease backgrounds, consistent with previous findings that mild, zwitterionic detergents preserve fibrillar collagens and maintain ECM integrity (Koziol-White et al., 2024; Leiby et al., 2022). Interestingly, we observed a slight but significant increase in thin collagen fibers after decellularization, especially in IPF samples. We theorize that the zwitterionic detergent SB-10 used during decellularization has a destabilizing/denaturing effect on the collagen fibers, resulting in less thick and more loosely organized collagen being detected by this method (Nandi et al., 1985). Another possible explanation might be altered birefringence or staining behavior after decellularization. Elastin was preserved in fibrotic scaffolds but reduced to approximately 60% in non-fibrotic tissue, which is only slightly above what other studies stated (Ott et al., 2008; Petersen et al., 2012; Dabaghi et al., 2021). One possible explanation for the discrepancy between the preservation of elastin in decellularized fibrotic and normal tissue could be the greater flushing of the tissue, as it is much more loosely organized tissue than the densely packed fibrotic tissue. This indicates that it might be advantageous to adapt the protocol to the density of the tissue from the different pathological background. A slightly shortened incubation time and agitation during the incubation with the zwitterionic detergent SB-10 will most likely reduce the disruption of the elastic fibers in normal lung tissue, while still being effective enough in removing the cellular material. All in all, the adapted protocol of an apoptosis-assisted decellularization was very effective in removing cells from the primary human lung tissue with a non-fibrotic and fibrotic pathological background, while greatly preserving the structural integrity and two of the main components (collagen and elastin) which are crucial for the investigation of matrix-cell feedback loops.

### 4.3 Functional recellularization and disease-specific responses

Two major challenges arise when repopulating decellularized lung scaffolds. First, scaffolds must be populated evenly to prevent cell clustering, as cells need to attach to the ECM rather than to each other for proper interaction studies. Second, cells often preferentially attach to the bottom of cultivation plates instead of the scaffolds, leading to bias in analysis - especially since fibroblasts proliferate more on stiff culture plates. We overcame this issue by usage of an anti-adherence solution and demonstrated successful colonization of SDLS with human primary lung fibroblasts (Figure 8). Interestingly, the differential contraction observed pronounced in non-fibrotic scaffolds but absent in fibrotic ones - supports the notion that our model retains functional disease-specific properties. It should be noted that this was only observed on a qualitative level and was not quantified. The reduced WST-1 activity of fibroblasts on SDLS compared to 2D plastic cultures (Figure 8H) aligns with previous reports that physiological ECM properties can suppress fibroblast hyperproliferation observed in rigid plastic environments (Discher et al., 2005; Hinz et al., 2001). Importantly, LDH cytotoxicity remained low (Figure 8G), indicating that decreased metabolic activity reflects a more physiological activation state rather than cell death (Thannickal et al., 2004).

### 4.4 Limitations and future perspectives

One limitation is the current need for small tissue fragments (∼0.5 cm^3^) to ensure full reagent penetration, potentially restricting applications requiring large continuous ECM areas (e.g., biomechanical testing) (Balestrini and Niklason, 2015). Scale-up may be possible using perfusion-based or agitation-enhanced systems. In our work, cytotoxicity values remained below ∼15% of the fully lysed positive control, in line with precedents in scaffold-based cultures where low LDH increase is interpreted as biocompatible (Biagini et al., 2021; Thurzo et al., 2022). In addition, similar LDH ranges have also been reported in previous work using long-term human precision-cut lung slice (hPCLS) cultures (Temann et al., 2017; Preuß et al., 2022), further supporting that the observed variability and viability levels are typical for primary human *ex vivo* lung models. The reduced WST-1 signal in fibroblasts cultured on SDLS likely reflects lower proliferative activity, as this assay primarily captures proliferation-associated metabolic turnover. Nonetheless, complementary transcriptomic or metabolomic analyses will be required to determine whether this behavior corresponds to a more quiescent or matrix-modulated phenotype. While the model itself preserves disease-specific ECM features and supports reseeding, the interpretation of our findings is limited by the heterogeneity of human tissue, the small sample numbers, and the fact that our analyses were restricted to end-stage fibrotic disease, which may not fully capture earlier disease stages. Furthermore, while initial reseeding experiments demonstrated the feasibility of colonizing SDLS, colonization efficiency was not yet quantitatively assessed. Additional optimization of seeding protocols and media composition will likely improve cell survival and enable more robust recellularization in future studies. Despite these limitations, the present approach provides a solid foundation for further methodological development and translational use. The potential applications of the SDLS platform are outlined below.

### 4.5 Applications and translational potential

Beyond its methodological advantages, the standardized SDLS format offers several opportunities for translational and mechanistic research. Disease-specific scaffolds enable direct comparison of ECM effects across physiological, IPF, and FHP backgrounds. Moreover, the standardized and agarose-free scaffold format facilitates efficient reseeding with primary human cells as demonstrated for fibroblast cultures, thereby providing a robust platform to study cell-matrix interactions in disease-specific ECM backgrounds. Importantly, the apoptosis-assisted decellularization approach used here combines biological and physicochemical cell removal mechanisms, which may better preserve ultrastructural and biochemical ECM integrity compared to purely detergent-based protocols. This gentle decellularization likely enhances accessibility of reagents through apoptotic dismantling, thereby facilitating efficient nuclear clearance even in dense fibrotic tissue while maintaining collagen and elastin architecture.

In terms of precise application, the SDLS model could possess multiple advantages for translational research, offering future perspectives and underlining the relevance and technical advancement of our model for the ILD research community. (i) Mechanistic studies can address how disease-specific ECM cues regulate fibroblast activation through mechanotransduction and metabolic pathways, including YAP/TAZ and MRTF-A signaling as well as ECM-dependent metabolic adaptations (Gilpin et al., 2014; Liu et al., 2015). Importantly, (ii) therapeutic testing such as screening of antifibrotic compounds (e.g., nintedanib, pirfenidone) in a physiologically relevant human ECM context could be facilitated, given the reproducibility and physiological relevance of the scaffold environment (Burgstal et al., 2017; Wollin et al., 2015; Maher et al., 2018), and could serve as an excellent comparison to human PCLS for observing single-cell-type-specific effects. Beyond fibroblast-focused applications, the SDLS system is compatible with epithelial and endothelial cell reseeding, offering opportunities to reconstruct more complex multicellular fibrotic microenvironments and to study epithelial–mesenchymal or endothelial–mesenchymal interactions under defined ECM conditions. Future work integrating biomechanical analyses such as atomic force microscopy or microindentation could directly link ECM preservation with stiffness-dependent fibroblast contraction and remodeling behavior. Ultimately, our model could facilitate (iii) integrative studies with high-content multiple analysis as in integration with histological, proteomic, biomechanical, and live-imaging assays on uniform scaffold sections to investigate overall advantages of scaffold models in fibrosis, in the lung and beyond. Complementary proteomic or biochemical analyses (e.g., LC-MS ECM profiling or hydroxyproline and elastin quantification) may further validate ECM preservation and expand the mechanistic interpretability of SDLS-based experiments. Of note, the approach might be adaptable to other organs, such as liver and kidney, where decellularization is challenging due to higher tissue density that is further increased during fibrosis (Karsdal et al., 2017). Together, these extensions emphasize the versatility and translational potential of the SDLS platform as a physiologically relevant human ECM model for mechanistic and preclinical antifibrotic research.

## 5 Conclusion

By introducing a standardized, agarose-free format of decellularized human lung tissue, this study provides the lung research community with a reproducible and disease-specific platform that bridges a critical methodological gap. Unlike previous approaches, the SDLS format preserves major structural proteins such as collagen and elastin across normal and fibrotic conditions, while being compatible with reseeding and downstream molecular analyses. Beyond its technical advantages, this model enables systematic dissection of how the fibrotic microenvironment shapes cellular phenotypes, thereby offering novel opportunities for mechanistic studies and pre-clinical anti-fibrotic drug testing. In doing so, it not only advances ECM research in ILDs but also establishes a versatile resource for broader applications in pulmonary biology and regenerative medicine.

## Data Availability

The original contributions presented in the study are included in the article/Supplementary Material, further inquiries can be directed to the corresponding author.
