# Supplementary material for: A novel agarose-free, standardized generation and versatile ECM characterization of decellularized scaffolds from normal and fibrotic human lung tissue
Source: Front Bioeng Biotechnol. 2026 Mar 10;14:1772891. doi: 10.3389/fbioe.2026.1772891 (PMC13008860; doi:10.3389/fbioe.2026.1772891)
Supplement: Supplementary file 1 [file Supplementaryfile1.docx]

Supplementary Material

# Supplementary Figures

**Supplementary Figure 1.** **Exemplary images of apoptosis induction in human primary lung tissue.** Apoptosis was induced with 20 μM Camptothecin and 10 μM Raptinal. Tissue sample sections were prepared before, 24h after and 48 h after induction and stained with the TUNEL in situ apoptosis kit. Apoptosis results into breakdown of DNA and is detected by the kit. Apoptotic cells appear in yellow. Cell nuclei were stained with DAPI and appear in blue. The positive control was prepared by incubating the tissue section with DNase. **A**: Exemplary images of the positive control, negative control and fresh and unprocessed tissue sections of normal and fibrotic lung tissue. **B**: Exemplary images of normal and fibrotic tissue samples that either incubated in culture medium or apoptosis induction medium for 24h and 48h.


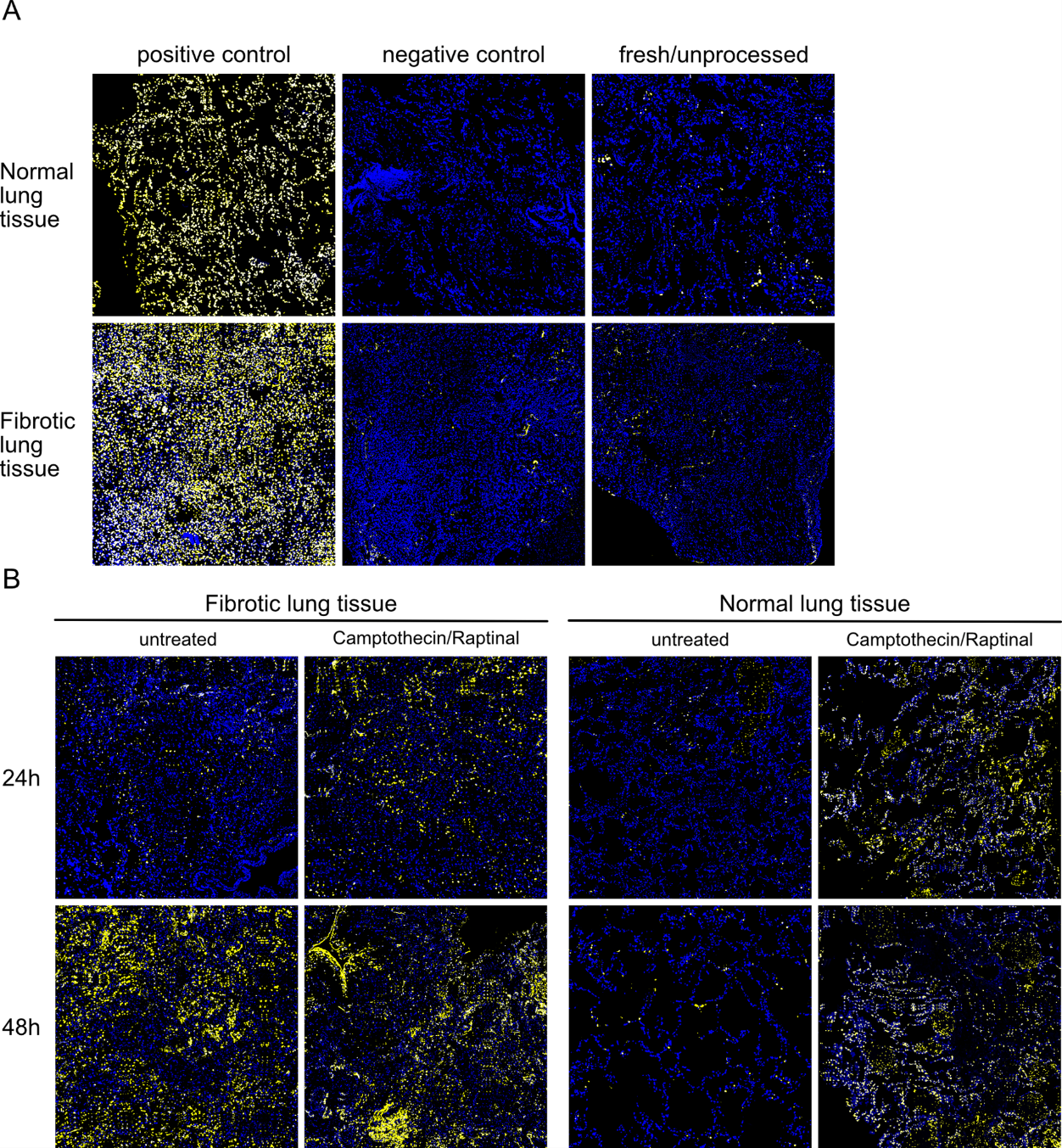


**Supplementary Figure 2. Analysis of collagen content in unprocessed and decellularized lung tissue.** The relative area of thick and thin collagen fibers was analyzed using polarized-light images of picro-sirius red (PSR) stained tissue sections. Under polarized light collagen fibers show a birefringence pattern, where thick collagen fibers appear in the spectrum of yellow and orange and thin collagen fibers appear greenish, while all other structures are no longer visible, allowing specific quantification of the relative area of collagen fibers of different sizes. **A:** An exemplary bright-field image of a PSR stained decellularized tissue section. Collagen fibers are stained in red **B:** The same field of view as seen in **A** but shown under polarized light. While thick collagen fibers appear in the spectrum of yellow and orange (**spiky arrows**), thin collagen fibers appear greenish (**hollow arrows**). **C:** Polarized-light image of a PSR stained decellularized tissue section with a color-threshold set for the specific detection of thick collagen fibers (**spiky arrows**). **D:** Overlay of the signal detected in the image from **C**. **E:** Polarized-light image of a PSR stained decellularized tissue section with a color-threshold set for the specific detection of thin collagen fibers (**hollow arrows**). **F:** Overlay of the signal detected in the image from **E**. Original magnifications: ×100.


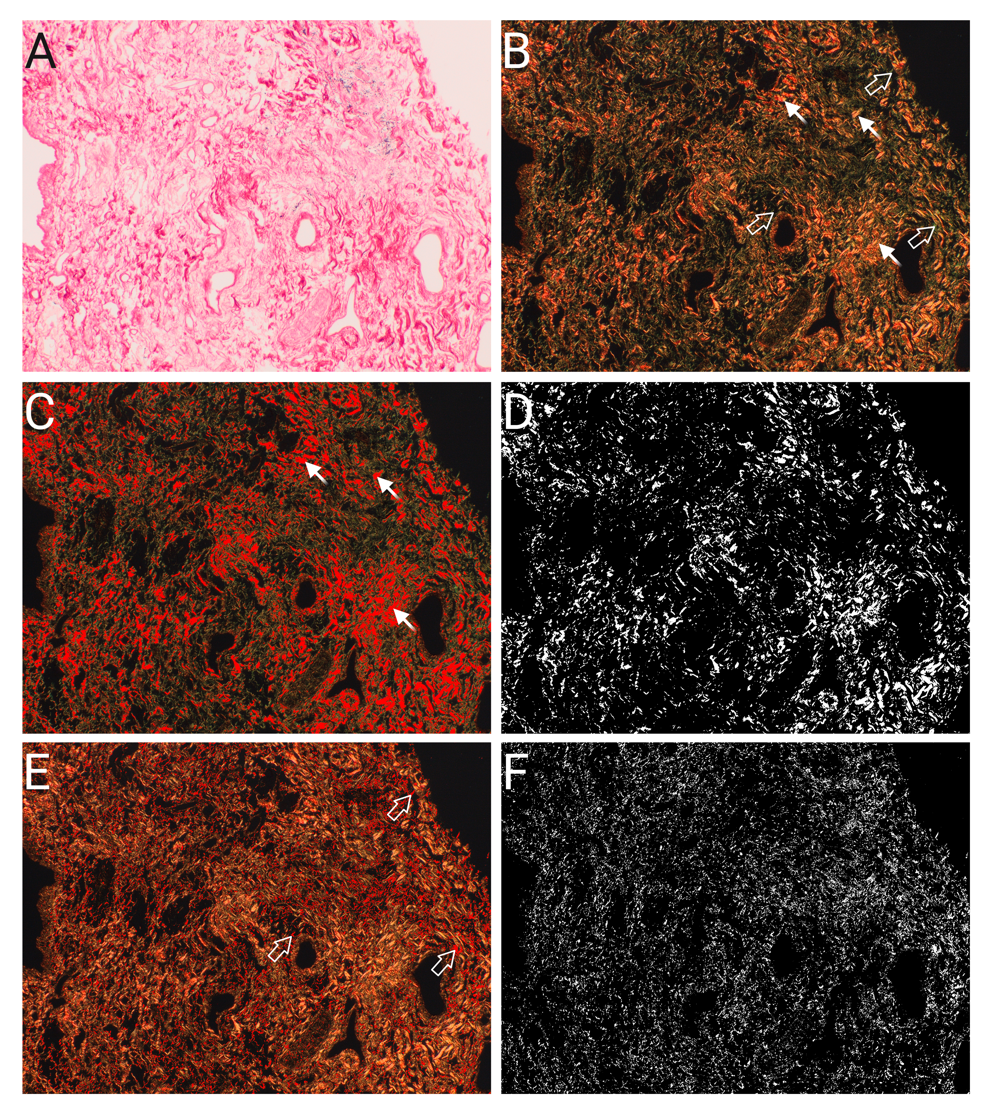


**Supplementary Figure 3.** **Analysis of the relative tissue area in unprocessed (fresh) and decellularized lung tissue samples.** The relative tissue area of each tissue section was quantified and used to compensate for different densities of the tissue samples and to exclude non-tissue areas like airspaces of healthy alveoli. **A**: Exemplary image of a whole tissue section of unprocessed (fresh) lung tissue in elastic Verhoeff-Van Gieson (EVG) stain. **B**: The same field of view (FOV) as in A, where the tissue area is selected (red overlay) using specific color threshold settings. **C**: Overlay of the signal detected in the image from B.


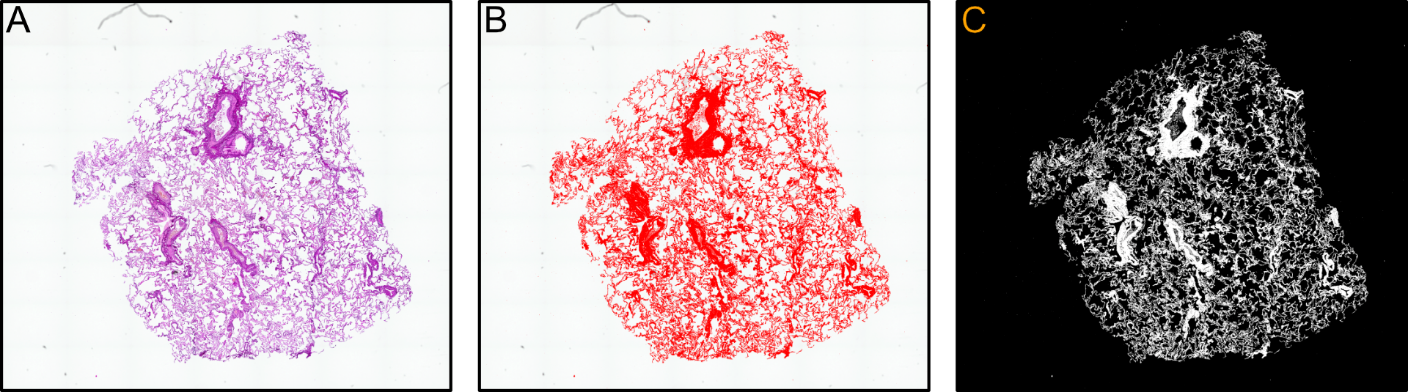


**Supplementary Figure 4.** **Testing of fibroblast repopulation of acellular lung scaffolds filled with agarose.** Agarose significantly hindered successful repopulation of the slices**.** Cells were not able to adequately enter the tissue and were forming cell clusters instead. Asterisks mark the agarose-filled airspaces of the tissue, and the arrows indicate cell cluster. **A:** Low-power stereoscopic image of an acellular, agarose-filled and repopulated lung slice. **B-C:** Low-power microscopic image (with H&E stain) of an acellular, agarose-filled and repopulated lung slice.


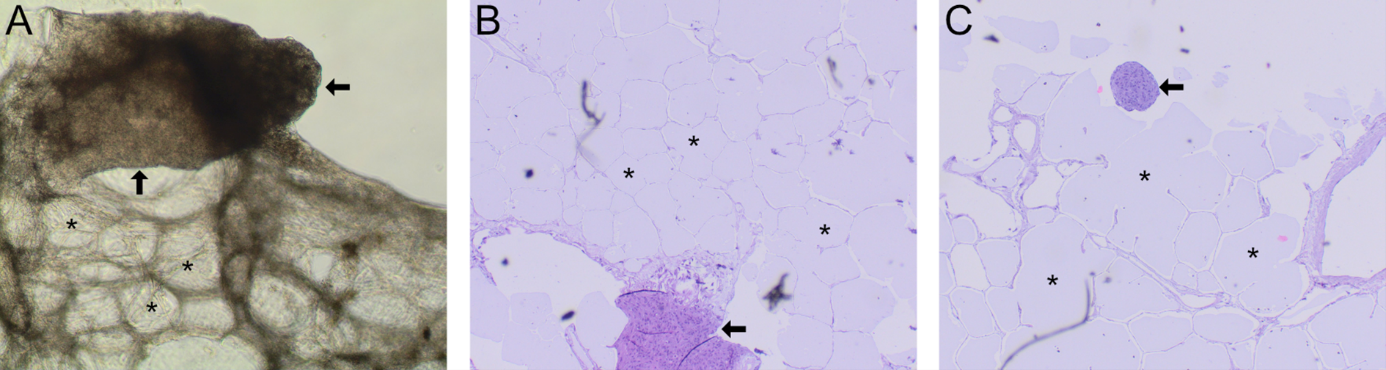


# Supplementary Tables

**Table S1. Patient cohort.**

| **ID** | **Usage** | **age [years]** | | **gender** | | **primary diagnostic finding** | **histologic evaluation of the sample** | **smoker status** |
| --- | --- | --- | --- | --- | --- | --- | --- | --- |
| N-1 | co-culture (SDLS fibroblasts) | 56 | | m | | pulmonary metastasis of an renal carcinoma | non-fibrotic with moderate emphysema | no |
| N-2 | dec. analysis, co-culture (SDLS) | 69 | | m | | pulmonary metastasis of an appendiceal carcinoma | non-pathological/normal | n/a |
| N-3 | dec. analysis, co-culture (SDLS) | 75 | | m | | pulmonary metastasis of a bladder carcinoma | non-pathological/normal | 25 py |
| N-4 | dec. analysis, co-culture (SDLS) | 23 | | w | | pulmonary metastasis of a rectum carcinoma | non-pathological/normal | n/a |
| N-5 | dec. analysis | 67 | | m | | Adenocarcinoma | non-fibrotic with minor emphysema | 50 py |
| N-6 | dec. analysis | 61 | | w | | Adenocarcinoma with moderate emphysema | non-fibrotic with moderate emphysema | n/a |
| N-7 | dec. analysis | 64 | | w | | PPEE and COPD | non-fibrotic with minor emphysema | 40 py |
| N-8 | dec. analysis | 57 | | m | | pulmonary metastasis of a colorectal carcinoma | non-fibrotic with minor emphysema | 20 py |
| N-9 | dec. analysis | 72 | | w | | Myzetoma with moderate emphysema | non-pathological/normal | 25 py |
| N-10 | dec. analysis , co-culture (fibroblasts) | 36 | | w | | pulmonary metastasis of a neuroendocrine neoplasia | non-pathological/normal | no |
| N-11 | dec. analysis , co-culture (fibroblasts) | 46 | | m | | Adenocarcinoma | non-pathological/normal | yes |
| IPF-1 | co-culture (SDLS& fibroblasts) | 60 | | m | | IPF | UIP pattern | yes |
| IPF-2 | dec. analysis , co-culture (SDLS & fibroblasts) | 61 | | m | | IPF | UIP pattern | n/a |
| IPF-3 | dec. analysis , co-culture (SDLS & fibroblasts) | 60 | | m | | IPF | UIP pattern | n/a |
| IPF-4 | dec. analysis, co-culture (SDLS) | 62 | | m | | IPF | UIP pattern | n/a |
| IPF-5 | dec. analysis | 63 | | m | | IPF | UIP pattern | yes |
| IPF-6 | dec. analysis | 60 | | m | | IPF | UIP pattern | 30 py |
| FHP-1 | co-culture (fibroblasts) | 62 | | w | | clinical: HP; histopathological: advanced interstitial fibrosis | UIP pattern | no |
| FHP-2 | dec. analysis , co-culture (fibroblasts) | 60 | | m | | clinical: HP; histopathological: advanced interstitial fibrosis | UIP pattern | yes |
| FHP-3 | dec. analysis | 66 | | m | | clinical: HP; histopathological: advanced interstitial fibrosis | UIP pattern | n/a |
| FHP-4 | dec. analysis , co-culture (SDLS & fibroblasts) | 60 | | m | | clinical: HP; histopathological: advanced interstitial fibrosis | UIP pattern | yes |
| FHP-5 | dec. analysis | 66 | | m | | clinical: HP; histopathological: advanced interstitial fibrosis | UIP pattern | no |
| FHP-6 | dec. analysis | 58 | | m | | clinical: HP; histopathological: advanced interstitial fibrosis | UIP pattern | no |
| FHP-7 | dec. analysis, co-culture (SDLS) | 59 | | m | | clinical: HP; histopathological: advanced interstitial fibrosis | UIP pattern | n/a |
| FHP-8 | dec. analysis | 61 | | m | | clinical: HP; histopathological: advanced interstitial fibrosis | UIP pattern | n/a |
| FHP-9 | dec. analysis, co-culture (SDLS) | 55 | | m | | clinical: HP; histopathological: advanced interstitial fibrosis | UIP pattern | n/a |
| FHP-10 | dec. analysis | 61 | | m | | clinical: HP; histopathological: advanced interstitial fibrosis | UIP pattern | no |
| UIP-1 | dec. analysis | 67 | | w | | unspecified entity (IPF or HP) with UIP pattern | UIP pattern | n/a |
| UIP-2 | dec. analysis | 65 | | m | | mixed fibrosis pattern (PPEE & UIP) | UIP pattern | n/a |
|  | | | | | | | | |
| **Average age per disease** | | | **age** | **SD** |  |  |  |  |
| IPF | | | 61 | 1 |  |  |  |  |
| FHP | | | 61 | 3 |  |  |  |  |
| normal | | | 57 | 16 |  |  |  |  |
| unclassified with UIP | | | 66 | 1 |  |  |  |  |
|  | | |  |  |  |  |  |  |
| **Total number of cases** | | | m | w |  |  |  |  |
| IPF | | | 6 | 0 |  |  |  |  |
| FHP | | | 9 | 1 |  |  |  |  |
| normal | | | 6 | 5 |  |  |  |  |
| UIP | | | 2 | 1 |  |  |  |  |

Demographical information and experimental usage of lung tissue utilized in this study; co-culture (fibroblasts), Isolation of fibroblasts and use for co-culture experiments; co-culture (SDLS), Generation of SDLS and use for co-culture experiments; dec. analysis, Decellularization of tissue samples and analysis of cell removal and ECM preservation; COPD, Chronic Obstructive Pulmonary Disease; PPFE, Idiopathic Pleuroparenchymal Fibroelastosis; IPF, Idiopathic Pulmonary Fibrosis; clinical: HP; histopathological: advanced interstitial fibrosis=FHP; UIP, Usual Interstitial Pneumonitis; n/a, not available; py, pack years.

**Supp. Table 2.** **Results from flow cytometry analysis of fibroblast isolations**.

|  | **sample** | **CD90** | **CD45** | **CD31** |
| --- | --- | --- | --- | --- |
|  | Isotype control | <1% | <1% | <1% |
| Normal/ non-fibrotic | N-F-1 | **98.6%** | <1% | <1% |
|  | N-F-2 | **98.9%** | <1% | <1% |
|  | N-F-3 | **95.6%** | <1% | <1% |
| Fibrotic hypersensitivity pneumonitis | FHP-F-1 | **98.9%** | <1% | <1% |
|  | FHP-F-2 | **99.8%** | <1% | <1% |
|  | FHP-F-3 | **97.4%** | <1% | <1% |
| Idiopathic pulmonary fibrosis | IPF-F-1 | **98.7%** | <1% | <1% |
|  | IPF-F-2 | **99.9%** | <1% | <1% |
|  | IPF-F-3 | **95.3%** | <1% | <1% |

Quantity of positive cells detected for each marker is shown in percent. Abbreviations: FHP=Fibrotic hypersensitivity pneumonitis, F=Fibroblasts, IPF=Idiopathic pulmonary fibrosis, N=Normal.

**Supplementary Methods**

**1. Analysis of successful cell nuclei removal in decellularized lung tissue**

Tissue samples were taken before and after decellularization. Histological sections were taken from the central part of those samples to evaluate both, the center as well as the peripheral area. Sections were stained with DAPI to identify cell nuclei. The stained sections were analyzed using a fluorescence microscope (APX100, Olympus Evident, Hamburg, Germany). The images were taken at 100x magnification at random locations on the tissue section using the DAPI channel (emission: 460/50) for nuclei staining and the GFP channel (emission: 510/40) to record the auto-fluorescence of the connective tissue fibers present in lung tissue sections. The number of cell nuclei present in each picture was analyzed using the merged channel image to exclude unspecific auto-fluorescence signal of connective tissue fibers. The images were processed with the image processing package Fiji, which is based on ImageJ2. Cell nuclei were specifically selected by adjusting the image color threshold to the following settings:

*Hue:* 150 - 255

*Saturation:* 0 - 255

*Brightness:* 80 – 255

Subsequently, the cell nuclei were counted using the “analyze particles” function of the software with the following settings:

*Size (inch^2):* 0.001 - infinity

*Circularity:* 0 - 1

*Exclude on edges:* no

*Include holes:* yes

The settings were integrated into a macro script to automatically analyze all images. The means of the technical replicates were calculated and used for data plotting and statistical analysis with GraphPad Prism v10.

**2. Analysis of ECM preservation in decellularized lung tissue**

Tissue samples were taken before and after decellularization. Histological sections were taken from the central part of those samples to evaluate both, the center as well as the peripheral area. Sections were either stained with hematoxylin and eosin (H&E), elastic Verhoeff-Van Gieson (EVG) or picro-sirius red (PSR). The stained sections were analyzed with an optical microscope. H&E is a standard histopathological staining that was used to evaluate the general structural preservation of the extracellular matrix (ECM). EVG stains elastic fibers in dark purple, which was used to specifically quantify the relative area of elastic fibers within the total tissue area. PSR stains collagens in red. When a polarization filter is applied, thick collagen fibers appear in the spectrum of yellow and orange and thin collagen fibers appear greenish. The image produced with the polarization filter was used to specifically quantify the relative area of thick and thin collagen fibers within the total tissue area. The images were processed with the image processing package Fiji, which is based on ImageJ2. The area of interest was specifically selected by adjusting the color threshold of the image to the following settings:

Elastic fibers in the EVG image:

*Hue:* 0 - 205 (fresh), or 0 - 197 (dec.)

*Saturation:* 0 - 255

*Brightness:* 0 - 175

The total tissue area of the EVG image:

*Hue:* 110 - 255

*Saturation:* 20 - 255

*Brightness:* 0 – 255

Thick collagen fibers in the PSR image (with polarization):

*Hue:* 0 - 255

*Saturation:* 0 - 255

*Brightness:* 165 – 255

Thin collagen fibers in the PSR image (with polarization):

*Hue:* 27 - 165

*Saturation:* 0 - 255

*Brightness:* 48 – 245

The total tissue area of the PSR image (without polarization):

*Hue:* 0 - 255

*Saturation:* 50 - 255

*Brightness:* 0 – 255

The settings were integrated into a macro script to automatically analyze all pictures. The data was collected in a Microsoft excel sheet. The means of the technical replicates were calculated and used for data plotting and statistical analysis with GraphPad Prism v10.

**3. Macro scripts for image processing**

A macro was created to analyze all pictures of the stained tissue sections automatically. The framework used for setting the color threshold of an image was as seen below. The values for “Hue”, “Saturation” and “Brightness” were adjusted for each staining to specifically select the area of interest. While the area of interest was measured using the "measure" function, the cell nuclei were measured using the “analyze particles” function. The macro was applied to all images using the “batch process” function of the software.

**Macro framework:**

*// Color Thresholder 2.9.0/1.53t*

*// Autogenerated macro*

min=newArray(3);

max=newArray(3);

filter=newArray(3);

a=getTitle();

run("HSB Stack");

run("Convert Stack to Images");

selectWindow("Hue");

rename("0");

selectWindow("Saturation");

rename("1");

selectWindow("Brightness");

rename("2");

min[0]=0;

max[0]=255;

filter[0]="pass";

min[1]=0;

max[1]=255;

filter[1]="pass";

min[2]=0;

max[2]=255;

filter[2]="pass";

**for** (i=0;i<3;i++){

selectWindow(""+i);

setThreshold(min[i], max[i]);

run("Convert to Mask");

**if** (filter[i]=="stop") run("Invert");

}

imageCalculator("AND create", "0","1");

imageCalculator("AND create", "Result of 0","2");

**for** (i=0;i<3;i++){

selectWindow(""+i);

close();

}

selectWindow("Result of 0");

close();

selectWindow("Result of Result of 0");

rename(a);

*// Colour Thresholding-------------*

run("Analyze Particles...", "size=0.001-Infinity clear include summarize");

*//or*

run("Measure");
